# Editorial: Multimodal approaches to investigating neural dynamics in cognition and related clinical conditions: integrating EEG, MEG, and fMRI data

**DOI:** 10.3389/fnsys.2025.1495018

**Published:** 2025-02-11

**Authors:** Golnaz Baghdadi, Fatemeh Hadaeghi, Chella Kamarajan

**Affiliations:** ^1^Biomedical Engineering Department, Amirkabir University of Technology (Tehran Polytechnic), Tehran, Iran; ^2^Institute of Computational Neuroscience, University Medical Center Hamburg-Eppendorf, Hamburg, Germany; ^3^Department of Psychiatry and Behavioral Sciences, SUNY Downstate Health Sciences University, Brooklyn, NY, United States

**Keywords:** brain studies, multimodal recording, EEG-MRI, fNIRS, EEG-fMRI, MEG, neurofeedback training (NFB), clinical data analysis

Cognitive processes, such as attention, memory, and decision-making, emerge from complex and dynamic interactions across multiple brain regions. Each brain imaging modality provides unique insights into different aspects of these intricate neural networks, allowing for a more comprehensive understanding of the underlying mechanisms.

Electroencephalography (EEG) measures the electrical activity of the brain by recording the postsynaptic potentials of neurons with electrodes on the scalp (Thio and Grill, 2023). Magnetoencephalography (MEG) detects the subtle magnetic fields generated by synchronized electrical currents from large neuronal assemblies as they communicate (Myllyla et al., 2017; Gross, 2019).

While magnetic resonance imaging (MRI) provides detailed information about internal structure of the brain, functional magnetic resonance imaging (fMRI) measures brain activity by detecting changes in blood flow associated with neuronal activity (Ebrahimzadeh et al., 2022). Functional near-infrared spectroscopy (fNIRS) also measures changes in the concentration of oxygenated and deoxygenated hemoglobin in cortical blood vessels, providing an indirect measure of brain activity (Chen et al., 2020; Providência and Margolis, 2022).

The Research Topic “*Multimodal approaches to investigating neural dynamics in cognition and related clinical conditions: integrating EEG, MEG, and fMRI data*” includes studies that use combined brain imaging methods such as EEG-MRI, MEG-MRI, and NIRS to better understand how the brain works in cognitive processes and diseases. The aim of these studies is to combine different imaging methods to increase the accuracy of brain measurements and obtain new information about brain function and disorders. This method helps us better examine the complex relationships between brain structure, function, and behavior. In particular, two studies examine the combination of EEG and MRI and show that this combination can give us more detailed and personalized information about brain activity and its disorders.

In this Research Topic, Tetsuka et al. utilized neurofeedback training to prevent cognitive decline in acute stroke patients. They monitored prefrontal activity using fNIRS and demonstrated that even short-term interventions can modulate neural activity and preserve cognitive function. Brain lesion locations in patients were identified using diffusion-weighted magnetic resonance imaging (DTI) or computed tomography (CT) scans. These regions were used as reference points to target specific brain areas in neurofeedback protocols and to assess the hemodynamic changes in those regions. This anatomical information can help personalize neurofeedback and other therapeutic methods. In addition to the neuroanatomical information, behavioral data including performance evaluations on a working memory task, were analyzed as a key indicator of the effects of neurofeedback and associated cerebral hemodynamic changes over time. The findings indicated that neurofeedback training was effective in enhancing prefrontal activity and maintaining cognitive function in acute stroke patients. The multidimensional approach enabled a thorough examination of the relationship between brain structure, function, and behavior.

Jones et al. conducted a detailed examination of EEG patterns during propofol-induced burst suppression in patients with treatment-resistant depression. Their study identifies distinct types of burst activity, highlighting the variability of neural responses to anesthesia. Participants were randomized to receive either high- or low-dose propofol infusions. The study identified three distinct types of burst activity, 1- broadband bursts, 2- spindles, and 3- low-frequency bursts, each varying significantly across subjects in terms of occurrence, spectral power, and spatial patterns across the scalp. Combining EEG with clinical data (e.g., drug dosages and patient characteristics) provided valuable insights into the individualization of anesthetic dosing and the diverse neural responses to propofol, potentially leading to more tailored treatment strategies in clinical practice.

In another study, Mertiens et al. investigated how Parkinson's disease (PD) affects resting state networks (RSNs) using MEG to measure phase-amplitude coupling (PAC). This study combined MEG with T1-MRI scans. MEG captured dynamic neural oscillations and PAC within RSNs, while T1-MRI provided accurate source reconstruction of the MEG signals. This integration allows for precise mapping of RSNs and revealed significant alterations in these networks in PD patients compared to healthy controls, particularly in the sensorimotor network (SMN). Levodopa medication significantly normalized SMN activity toward a state similar to that of healthy controls, though no significant changes were observed in the optimal PAC coupling frequencies, suggesting that levodopa primarily affects motor symptoms without influencing all RSNs in the same way.

The results of the three above-mentioned studies demonstrate how combining different modalities can enhance treatment process and provide deeper insights into brain diseases and disorders. However, as previously discussed, integrating multiple modalities present challenges, and some studies are focused on improving the integration of these modalities and addressing technical issues.

Karittevlis et al. introduce a novel method for analyzing EEG and MEG data of evoked responses to somatosensory stimulation, aimed at identifying early responses in the thalamus and cortex. EEG-MEG studies often face challenges in accurately determining brain activity due to the reliance on complex models with many assumptions. These models can be particularly inaccurate for EEG because the skull distorts electrical signals. Additionally, variability in responses to the same stimulus across trials complicate the achievement of consistent results. The study proposes a straightforward approach that bypasses complex modeling and uses a method called virtual sensors (VS), which combines EEG and MEG data to directly capture brain activity. This method improves the accuracy of elicited brain activity and identifies trial-to-trial variability, offering clearer and more reliable insights into the communication between different brain regions. By simplifying the computational processes, the authors present a method with potential applications in real-time neurofeedback and brain-computer interfaces, highlighting the practical benefits of multimodal approaches in applied neuroscience.

The study conducted by Dayarian and Khadem leverages multimodal recording by integrating EEG with MRI-based head models to introduce a new algorithm for EEG source localization. Accurate EEG source localization is crucial in clinical applications for diagnosing and planning treatments for neurological disorders such as epilepsy, by precisely identifying the location of abnormal brain activity. It also plays a key role in surgical planning, helping to minimize damage to critical brain regions, and aids in in monitoring disease progression and tailoring treatments to individual patients, thus enhancing personalized medicine (Yang et al., 2023).

However, EEG source localization faces several challenges due to the complex geometry of the head and brain, which complicates accurate modeling. Traditional methods often struggle with the heterogeneous properties of brain tissues and the low spatial resolution of EEG, making it difficult to differentiate closely situated sources. Additionally, EEG signals are susceptible to noise and artifacts, such as muscle activity or eye movements, which can obscure the true brain signals and hinder precise localization (Michel and Brunet, 2019; Hirata et al., 2024). Many techniques also rely on simplifying assumptions about the head and brain models, which may not accurately reflect individual anatomical variations, further impacting localization accuracy (Hirata et al., 2024). Accurate co-registration of EEG sensors with the head model is critical, as any misalignment can significantly affect the results.

Dayarian and Khadem propose a hybrid method that combines the strengths of the boundary element method (BEM) and finite element method (FEM), to address EEG source localization challenges. BEM effectively models isotropic brain regions and dipolar sources, while FEM accurately handles complex, anisotropic tissues in the head with greater accuracy. By validating this hybrid approach using MRI-based realistic head models, the study achieves significant improvements in accuracy for EEG forward problem solutions. The method demonstrates enhanced performance in error metrics compared to using FEM alone, highlighting the value of incorporating detailed anatomical information from MRI to improve the precision of neuroimaging analyses.

Each brain imaging method has its own advantages and limitations, as outlined in Table 1. To study fast dynamics, methods with high temporal resolution are essential to accurately capture rapid changes in brain activity. Among non-invasive brain imaging techniques, EEG and MEG stand out for their millisecond-level temporal resolution, making them ideal for studying fast cognitive processes. In contrast, fMRI and fNIRS offer temporal resolution on the order of seconds, which limits their applicability for investigating rapid brain dynamics.

**Table 1:** A summary of the features of each neuroimaging method.

**Method**	**Advantages**	**Disadvantages**
EEG	- High temporal resolution - Direct measure of neuronal activity - Relatively low cost - Non-invasive - Portable	- Low spatial resolution - Excessive artifacts - Limited depth penetration
MEG	- High temporal resolution - Direct measure of neuronal activity - Better spatial resolution than EEG	- Expensive - Limited to specialized facilities - Sensitive to magnetic noise
fNIRS	- Portable - Allows natural movement - Non - invasive - Easy to use	- Low spatial resolution - Limited depth penetration - Sensitive to scalp hemodynamics - Requires baseline
fMRI	- High spatial resolution - Whole - brain coverage - Can study deep brain structures	- Low temporal resolution - Expensive - Non-portable - Requires stillness from subjects

In terms of spatial resolution, the most sensitive methods are fMRI, followed by fNIRS, MEG, and EEG. High spatial resolution is crucial for accurate brain mapping. fMRI offers the highest spatial resolution (in millimeters), allowing for visualization of active brain regions, including deeper layers. In contrast, EEG has the lowest spatial resolution. Although source localization methods have been developed to mitigate this limitation, they face challenges due to volume conduction and require a high density of EEG electrodes to accurately determine the source of brain activity (Liu et al., 2023).

Apart from the challenges of temporal and spatial resolution, factors such as usage complexity, portability, and cost are also crucial considerations when se Apart from the challenges of temporal and spatial resolution, factors such as usage complexity, portability, and cost are also crucial considerations when selecting a neuroimaging method.

To leverage the advantages of various brain imaging methods and address the limitations of each technique, multimodal recording is recommended. However, the simultaneous use of two or more different techniques presents several challenges, which are detailed in the following sections.

## 1 EEG and fMRI

The primary advantage of EEG-fMRI co-recording studies is their ability to provide both high temporal resolution (through EEG data) and high spatial resolution (through fMRI data), resulting in a more comprehensive understanding of brain function and cognitive processes. However, EEG-fMRI studies face several challenges. One significant issue is the need for MR-compatible EEG hardware, which can increase costs. MR-compatible systems are designed to be non-magnetic and to minimize induced voltages and currents that could arise from radiofrequency and gradient fields during MRI scans (Mele et al., 2019; Scrivener, 2021; Warbrick, 2022). If these voltages and currents are not adequately controlled, they can cause heating of the EEG or ECG leads, potentially posing risks to the participants (Kugel et al., 2003). To mitigate these risks, careful design of the wires and the use materials resistant to electrical currents or incorporating built-in resistors are necessary. In addition, the magnetic fields in the MRI environment affect the quality of EEG signals, reduce the signal-to-noise ratio, and increase post-processing time. Although EEG electrodes can also influence the MRI's magnetic fields, this effect is generally minimal and often disregarded. EEG-fMRI studies are typically conducted while participants are lying down, which may not be suitable for studies requiring participants to be in different positions, such as sitting, standing, or walking. Moreover, the MRI environment's cold temperatures and intense noise can be uncomfortable and may influence brain activity, especially in children and the elderly, potentially confounding the results. Lastly, EEG-fMRI studies are not well-suited for investigating sleep and its disorder due to the discomfort of the MRI environment and the impracticality of extended scanning sessions (Duyn, 2012; Mele et al., 2019; Ebrahimzadeh et al., 2022).

Analyzing EEG and fMRI data from simultaneous recordings presents unique challenges and requires specific methods. Common approaches to data analysis in EEG-fMRI studies include (Huster et al., 2012; Abreu et al., 2018):

1. Symmetrical approaches
  - Model-based techniques: these involve creating and using mathematical models to understand the relationship between EEG and fMRI data.
  - Data-driven techniques: methods such as independent component analysis (ICA) and canonical correlation analysis (CCA) are used to extract and correlate components from EEG and fMRI data.
2. Asymmetrical approaches
  - fMRI-Driven EEG: this approach uses fMRI data to inform and enhance the interpretation of EEG signals.
  - EEG-Informed fMRI: conversely, EEG data can be used to guide and refine fMRI analysis.
3. Data quality and artifact correction
  - Gradient artifacts (GA): artifacts caused by the MRI gradient fields that can distort EEG data.
  - Pulse artifacts (PA): artifacts resulting from the MRI scanner's pulse sequences that can interfere with EEG recordings.
  - Correction techniques
    ° Time-domain subtraction: a method to subtract artifacts based on time-domain signals.
    ° Independent component analysis (ICA): a technique used to isolate and correct artifacts by decomposing the data into independent components.
    ° Hardware solutions: implementing specialized hardware to minimize or correct artifacts.

These methods are essential for addressing the complexities of integrating EEG and fMRI data and ensuring the accuracy of the results. Despite the mentioned challenges, EEG-fMRI studies are more frequently used than other multimodal approaches. This preference is likely due to the maturity of these technologies and their relatively lower cost compared to other multimodal systems.

## 2 EEG and fNIRS

While fMRI offers high spatial resolution, it has notable limitations such as lack of portability, inability to record in sitting or standing positions, restricted recording duration, and an environment that is cold and noisy. Functional near-infrared spectroscopy (fNIRS) addresses these limitations by measuring brain metabolic or hemodynamic activity similarly to fMRI but with greater flexibility. Although fNIRS has relatively lower spatial resolution and its accuracy can be influenced by individual differences such as skin color and hair type, it is well-suited for recording in various positions (sitting, standing, or walking) and over extended periods. Consequently, EEG-fNIRS provides an effective solution for combining both temporal and spatial resolution without the concerns of portability and MR-compatible EEG systems. Moreover, fNIRS offers advantages such as lower cost and smaller maintenance requirements (Liu et al., 2021; Uchitel et al., 2021).

One technical challenge in the simultaneous EEG and fNIRS recording is configuring the sensors related to two modalities for minimal interference. Ongoing technological development are needed to improve the integration of these modalities (Ahn and Jun, 2017; Chen et al., 2020; Li et al., 2022).

Pre-processing and processing for each modality involve specific steps, but simultaneous recording introduces additional challenges, including the potential negative impact of one modality on the other and the integration of data from both modalities. The following solutions address these challenges (Hossain et al., 2022; Li et al., 2022; Mughal et al., 2022):

1. Data synchronization
  - Clock synchronization or temporal alignment
2. Artifact removal
  - Shared artifacts (e.g., motion artifacts)
  - Physiological artifacts (e.g., heartbeat, respiration)
3. Signal filtering
  - Bandpass filtering for EEG
  - Spatial filtering for fNIRS
4. Motion artifact correction
  - Simultaneous correction (e.g., wavelet-based)
  - Correlation-based methods
5. Baseline correction
  - Common baseline establishment
  - Global signal regression for fNIRS
6. Data integration
  - Feature extraction (e.g., EEG alpha power, fNIRS HbO/HbR concentrations)
  - Joint analysis (e.g., canonical correlation analysis, joint ICA)
7. Statistical analysis
  - Cross-modality correlation or multimodal data fusion

This approach facilitates a more comprehensive analysis and integration of EEG and fNIRS data, thereby improving the overall understanding of brain function.

## 3 MEG and fMRI

Simultaneous use of MEG and fMRI recording can achieve both high temporal and spatial resolution. However, integrating these two modalities presents significant challenges, including the high cost of both techniques, the necessity for MR-compatible MEG systems, and the adverse effects of fMRI magnetic fields on MEG recordings. To mitigate these challenges researchers can employ several strategies (Im et al., 2005; Plis et al., 2010; Hall et al., 2014; Cichy et al., 2016):

1. Minimize temporal overlap: reduce the concurrent time of MEG and fMRI data to lessen interference.
2. Use precise timing triggers: ensure accurate alignment of MEG and fMRI recordings with precise timing triggers.
3. Shield MEG sensors: protect MEG sensors from external electromagnetic interference.
4. Apply ICA: use ICA to separate fMRI noise from MEG signals.

These strategies help address the technical difficulties of combining MEG and fMRI, facilitating more effective integration of their data.

## 4 MEG and fNIRS

To address the limitations of the MEG-fMRI co-recording, replacing fMRI with fNIRS can be advantageous. This substitution leverages the benefits of MEG and fNIRS while reducing some of the associated challenges.

Despite MEG's higher spatial resolution and superior signal-to-noise ratio compared to EEG, simultaneous use of EEG and fNIRS is more common than MEG-fNIRS due to the high cost and complex maintenance of MEG systems. MEG-fMRI is also used more frequently than MEG-fNIRS. The technical difficulty in achieving simultaneous recordings with MEG and fNIRS arises from the differing physical requirements of each modality. For instance, the spatial configuration for fNIRS optodes necessitates specific placement that may not align optimally with the measurement points for MEG sensors (Ru et al., 2022).

To effectively analyze and integrate MEG and fNIRS data, specific preprocessing and fusion methods are recommended:

1. Temporal alignment: synchronize MEG and fNIRS data to a common time reference.
2. Artifact removal and noise reduction
  - MEG: apply temporal filtering to remove drifts and high-frequency noise, and use ICA to address eye and muscle artifacts.
  - fNIRS: perform baseline correction and detrending to manage slow drifts and noise, and use wavelet or adaptive filtering to handle motion and physiological noise.
3. Spatial alignment: align the spatial coordinates of MEG and fNIRS data to a common anatomical reference.
4. Data standardization: ensure comparability by standardizing MEG and fNIRS data.
5. Feature extraction and fusion: extract relevant features from both datasets and combine them using advanced fusion techniques such as canonical correlation analysis (CCA), joint independent component analysis (jICA), and multimodal fusion algorithms.

These methods enable effective integration of MEG and fNIRS data, thereby enhancing the analysis and interpretation of combined datasets.

## 5 Brain imaging and behavioral data

Along with these methods, recording and analyzing behavioral data is also crucial for understanding cognitive functions and the brain processes. Behavioral data includes information about a person's performance in cognitive and psychological tasks, response time, and accuracy. These data enable researchers to correlate brain activity results with observed behaviors, providing a deeper understanding of how information is processed in the brain. Combining behavioral data with imaging and electrophysiological methods can reveal complex patterns of brain-behavioral interactions and offer a more comprehensive view of cognitive functions.

Thus, the choice of methods and their integration with behavioral data depends on the research type and objectives. Table 2 presents the benefits and challenges of various multimodal recording approaches.

**Table 2:** A summary of the benefits and challenges of multimodal recording methods.

**Combination**	**Benefits**	**Challenges**
EEG-fMRI	- Combines high temporal resolution of EEG with high spatial resolution of fMRI	- Technical complexity (need MR-compatible EEG) - Artifact correction is challenging - Expensive and non-portable
EEG-fNIRS	- Portable - Combines temporal resolution of EEG with hemodynamic information from fNIRS - Allows natural movement	- Limited spatial resolution - Artifact correction is challenging - Sensitive to noise
MEG-fMRI	- Combines high temporal resolution of MEG with high spatial resolution of fMRI	- Extremely expensive - Requires specialized facilities - Artifact correction is challenging
MEG-fNIRS	- Combines temporal resolution of MEG with hemodynamic information from fNIRS	- Low spatial resolution - Artifact correction is challenging - Limited to superficial brain activity

In summary, as multimodal approaches continue to advance, they are likely to be pivotal in developing more effective diagnostic and therapeutic strategies for psychiatric and neurological disorders. Future research should focus on improving computational methods for data integration and analysis, optimizing sensor configurations, and developing more robust techniques to mitigate artifacts. Such advancements could further deepen our understanding of brain function and pathology, offering new opportunities for research in cognitive neuroscience and clinical applications. Notably, while some review articles cover each modality combination separately, this Research Topic of original research offers unique empirical insights that enhance its value.
